# A Human Lymph node-on-a-Chip for Personalized Evaluation of Vaccine Immunogenicity

**DOI:** 10.21203/rs.3.rs-8951191/v1

**Published:** 2026-03-30

**Authors:** Fanghao Shi, Dongdong Liu, Yujing Song, John Manoyan, Tiffany Chan, Kesi K. Wilson, Anna Eichinger, Boris Reizis, Christopher Y. Park, Anoma Nellore, Katsuo Kurabayashi, Ralf Duerr, Ramin Sedaghat Herati, Weiqiang Chen

**Affiliations:** 1 Department of Mechanical and Aerospace Engineering, Tandon School of Engineering, New York University, Brooklyn, NY 11201, USA.; 2 Department of Biomedical Engineering, Tandon School of Engineering, New York University, Brooklyn, NY 11201, USA.; 3 NYU Langone Vaccine Center, Department of Medicine, New York University School of Medicine, New York, NY 10016, USA.; 4 Department of Pathology, New York University School of Medicine, New York, NY 10016, USA.; 5 Department of Microbiology, New York University School of Medicine, New York, NY 10016, USA.

## Abstract

Vaccines have revolutionized public health, yet their development remains hampered by the poor predictive power of animal models, leading to high clinical failure rates and variable efficacy across populations. To bridge this translational gap, we developed a human lymph node-on-a-chip model that biomimetically reproduces key physiological features of human lymph node, including compartmentalized immune cell zones and functional stromal networks. This immunocompetent model recapitulates the complete cascade of vaccine-induced adaptive immune processes in human lymph nodes, from antigen presentation, immune cell differentiation to germinal center formation and antibody secretion, providing a human-relevant platform for preclinical vaccine testing. Using donor-matched immune cells from influenza vaccine trials, we established a personalized clinical-trial-on-chip platform that accurately mirrors individual vaccine responses. Benchmarking on-chip immunogenicity readouts against clinical serological and transcriptomic data confirmed the platform’s predictive power for assessing vaccine efficacy across diverse populations. Our study uncovered a key mechanism of vaccine failure in vulnerable populations: age- and comorbidities-related factors impair T follicular helper cell differentiation and disrupt critical T-B cell interactions. Single-cell transcriptomic profiling revealed critical immune signaling networks involving MyD88 in DCs, IL-2/STAT5 balance in T cells, and TACI/BCMA activation in B cells that collectively govern the efficiency of the adaptive immune cascade. These mechanistic insights enabled us to validate clinically actionable strategies, including dose escalation and IL-2 cytokine adjuvant as effective countermeasures to enhance immunogenicity efficiency in immunocompromised individuals. These results demonstrate the potential of this human lymph node-on-a-chip as a transformative precision vaccinology tool for personalized vaccine immunogenicity assessment and optimization.

## INTRODUCTION

Vaccines have revolutionized public health by preventing infectious diseases and mitigating pandemics (1). However, the development of safe and effective vaccines remains constrained by the poor translational fidelity of preclinical animal models, stemming both from interspecies immunological divergence and human immune heterogeneity (2). In clinical practice, vaccine efficacy is primarily evaluated by measuring serum antibody titers, which lack mechanistic information about the immunogenic potency of vaccines (3, 4). The lymph node serves as the critical nexus for vaccine-induced adaptive immunity (5), where spatially organized microenvironments (B cell follicles, paracortical T zones, stromal conduits) coordinate the immunological cascade: from dendritic cell (DC) antigen capture and presentation to lymphocyte priming, germinal center (GC) maturation, and ultimately antibody secretion (6). However, this delicate immunogenic process is vulnerable to dysfunction in aging and comorbid populations, manifesting as T cell exhaustion, disrupted T-B cell interaction, attenuated GC reactions, and fibrotic stromal remodeling which collectively compromise vaccine responsiveness (7). Despite clear clinical correlates of variable efficacy, the mechanistic basis of these immune impairments remains unresolved, constrained by the absence of human model systems that capture interindividual variation in vaccine immunogenicity responses, a critical gap limiting the development of efficacious vaccines and precision vaccination strategies tailored to vulnerable populations.

The recent U.S. Food and Drug Administration’s (FDA) Modernization Act 2.0 marks a significant shift in preclinical drug development from traditional animal testing to human-relevant new approach methodologies (NAMs), such as *in vitro* organoids and organ-on-a-chip systems to enhance drug safety and efficacy predictions. However, to date, no *in vitro* model system has been able to simulate the whole human lymph node microenvironment and the complete dynamic process of adaptive immunity. In recent years, human tonsil and peripheral blood mononuclear cell (PBMC) derived lymphoid organoid models have been developed for the studies of human adaptive immune responses, such as studying vaccine-induced B cell maturation, plasma cell differentiation and antibody production (8–10). While lymphoid organoid cultures can partially replicate organ complexity, the lack of physiologically relevant three-dimensional (3D) anatomical structures such as the compartmentalized immune cell zones, functional stromal networks and vasculature compromise their biomimicry to their *in vivo* counterparts (11, 12). Recently developed microfluidic models have enabled *in vitro* study of lymph node function, focusing on biophysical cues such as subcapsular flow (13), lymphocyte migration behaviors (14), antigen-induced IgG secretion and follicle formation (15). However, existing systems largely rely on murine cells (16) or immortal human cell lines (17), and capture isolated features rather than fully reconstituting the complex, compartmentalized lymph node niche. As a result, they fail to capture the complete and spatiotemporal coordination of vaccine immunogenic events in individuals for translational vaccine assessment.

Here, we report a microphysiological lymph node-on-a-chip (LN-on-a-chip) model that biomimetically reproduces human lymph node physiology, including the spatially organized immune cell zones, 3D stromal networks, and vaccine-responsive immunogenicity in individuals. The scarcity of human lymphoid samples has constrained the development of physiologically relevant *in vitro* models (18). Recent studies suggest that blood-derived lymphocytes offer a promising alternative, have potential to ectopically form lymphoid-like structures, undergo antibody class switching, and generate plasma cells *in vitro* under inflammatory conditions (10, 15, 19). Using PBMCs from participants in influenza vaccine trials, we established a personalized ‘clinical-trial-on-chip’ platform that enables for a precise and individualized evaluation of vaccine responses across diverse populations. This immunocompetent model represents a transformative advancement in preclinical vaccine immunogenicity testing by recapitulating the complete cascade of vaccine-induced immune processes in human lymph nodes, from antigen presentation to T cell activation, plasmablast differentiation, GC formation, and antigen-specific antibody production. Mechanistically, we found age- and comorbidities-related factors induced severe disturbance of immune cell priming in lymph nodes, explaining the poor vaccine outcomes observed clinically in vulnerable populations. The platform further enables validation of clinically actionable strategies including dose escalation and cytokine adjuvants as effective countermeasures to enhance vaccine immunogenicity in immunocompromised individuals. Our human lymph node-on-a-chip thus holds a great translational potential for the development of more effective and safer vaccines for a broader population.

## RESULTS

### Reconstruction of human lymph node niche in an organotypic lymph node-on-a-chip model

**Extended Data Fig. 1.**
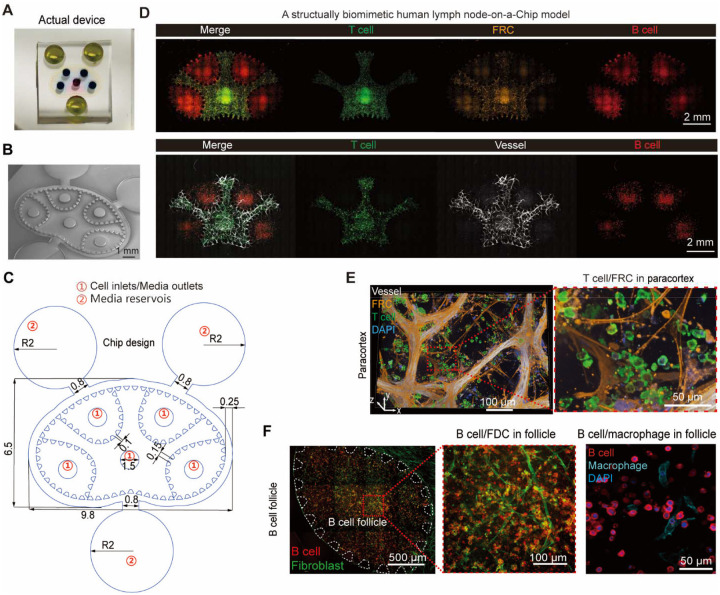
Biomimetic human lymph node-on-a-chip recapitulates tissue-like architectures. (**A**) Photograph of the assembled PDMS device, with central cell loading ports (black) and peripheral media reservoirs filled with dye (yellow). (**B**) SEM image of the chip master mold. (**C**) Design layout of the microfluidic device (footprint ≈ 9.8 × 6.6 mm) with interconnected central paracortex and surrounding B cell follicles compartments. Trapezoidal microposts retain hydrogels while permitting molecular exchange between compartments. The chip also contains five cell loading/effluent ports (①; D = 1.5 mm) and three peripheral media reservoirs (②; R = 2 mm). (**D**) Whole-scan immunofluorescence images of the chip showing spatial segregation of immune and stromal compartments. Top: CD3^+^ T cells, Phalloidin^+^ FRC reticulum and CD19^+^ B cells. Bottom: CD3^+^ T cells, CD31^+^ endothelium cells, and CD19^+^ B cells. (**E**) Immunofluorescence images of the paracortical stromal niche showing CD31^+^ HEV, PDPN^+^ FRC, and CD3^+^ T cell embedded in ECM. (**F**) Immunofluorescence images showing the follicular stromal niche (boxed region, higher magnification) with CD19^+^ B cells over a phalloidin-labeled FDC reticular scaffold, and immune niche with CD19^+^ B cells with CD163^+^ macrophages and nucleus (DAPI). For D-F, immunofluorescence images were taken on day 5 after vaccination.

**Fig. 1.**
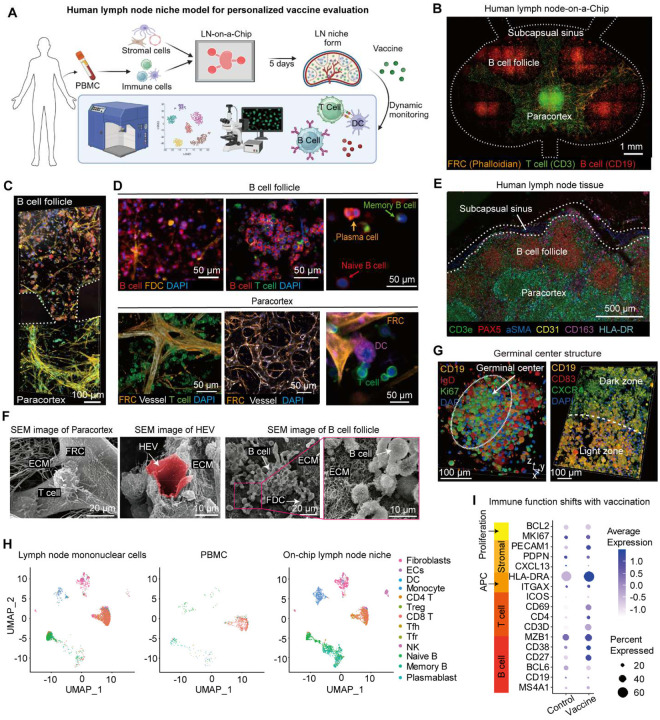
An organotypic and immunocompetent lymph node niche model. (**A**) Schematic of LN-on-a-chip for personalized vaccine evaluation. (**B**) Whole scan and (**C**) immunofluorescence image of the on-chip lymph node niche on day 5, showing the paracortical (CD3^+^ T cell, green; phalloidin^+^ FRC, orange) and follicular (CD19^+^ B cell, red; phalloidin^+^ FDC, orange) regions. (**D**) Immunofluorescence images showing key niche cells in the model. Top row (day 10): CD19^+^ B cells, CD55^+^ FDC, CD4^+^ T cells, naïve (CD19^+^CD27^−^CD138^−^), memory (CD19^+^CD27^+^CD138^−^), and plasma (CD19^+^CD27^−^CD138^+^) B cells. Bottom row (day 5): CD31^+^ vessel, Phalloidin^+^ (middle)/PDPN^+^ (right) FRC, CD11c^+^ DCs, CD3^+^ T cells. (**E**) Immunofluorescence image of a human mesenteric lymph node tissue, showing spatial organization of T cells (CD3ε), B cells (PAX5), mural/stromal cells (αSMA), endothelial cells (CD31), macrophages (CD163), and antigen-presenting cells (HLA-DR). (**F**) SEM images showing key niche structures on day 10. (**G**) GC-like structure at day 10 post-vaccination (left), with CD19^+^ B cells, IgD^+^ naïve B cells, Ki67^+^ proliferating B cells, and nuclei (DAPI). The right image shows GC zonal organization at day 12 post-vaccination, with CD19^+^ B cells, CD83^+^ GC B cells in light zone and CXCR4^+^ cells in dark zone. (**H**) Single-cell transcriptomic mapping using UMAP of human lymph node tissue (left), PBMC (middle), and LN-on-a-chip cultures (right) at day 10 post-vaccination. (**I**) Comparison of transcriptional activation on chip with or without (control) vaccination.

To model the human lymph node microenvironment and adaptive immune response in vitro, we established an organotypic and immunocompetent human LN-on-a-chip microphysiological system (Fig. 1A). Anatomically, lymph nodes are highly structured secondary lymphoid organs with a highly organized microenvironment comprising compartmentalized T cell zone (paracortex) and B cell follicles, specialized fibroblastic reticular networks and vascular structures that collectively coordinate adaptive immune responses (6). To replicate this native tissue architecture *in vitro*, the microfluidic model (Fig. 1B,C and **Fig. S1**) is constructed with the two major functional immune niche compartments of lymph nodes, the paracortex and the B cell follicles, with autologous human PBMC-derived lymphocytes supported with stromal reticulum of fibroblastic reticular cells (FRCs), follicular dendritic cells (FDCs), high endothelial venules (HEVs) and biomimetic extracellular matrix (ECM). In addition to these cellular compartments, the chip integrates surrounding microfluidic channels mimicking subcapsular sinus, afferent vessels and efferent vessels that allow perfusion of fluids and antigens into the lymph node niche (6). The microfluidic chip with functional compartment zones partitioned by regularly spaced micropillars was fabricated using replica molding of polydimethylsiloxane (PDMS) on a glass coverslip, following our previous protocols (Extended Data Fig. 1A–C) (20). Then the paracortex compartment was populated with human DCs, T cells, FRCs and endothelial stromal cells, the B cell follicle regions were populated with B cells and FDCs within a hydrogel ECM composed of fibrin, collagen, and Matrigel to build the microanatomic organization of lymph nodes (Fig. 1C, D). Spatially patterned seeding, followed by a culture in a custom medium formulation, enabled cells to self-organize into anatomical T cell-rich paracortex regions and B cell follicles embedded in biomimetic stroma structures, with cell viability maintained for over 15 days.

We first benchmarked the biomimicry of the LN-on-a-chip model against native human lymph node tissue by immunofluorescence imaging and single-cell RNA sequencing (scRNA-seq). The localization of CD3^+^ T cells, CD11c^+^ DCs and PDPN^+^ FRCs in the paracortex, CD19^+^ B cells and CD55^+^ FDCs (FDC-like YK6-CD40L-IL21 cell) in the B cell follicle region were determined by immunostaining (Fig. 1D), proving successful replication of lymph node paracortex, B cell follicle compartments. Stromal cells, including specialized endothelia cells, FRCs and FDCs formed reticular microenvironments within the lymph node chip to support immune cell functions in specific zones of the lymph nodes (21). In the paracortical region, endothelia cells (CD31^+^) self-organized into vascular networks resembling HEVs-like network, while FRCs (PDPN^+^) formed a fibroblastic reticular meshwork (Extended Data Fig. 1D–F) and deposited ECM components: laminin, collagen IV, and fibronectin over time, supporting T cell migration and HEV development (**Fig. S1**) (22). SEM imaging of the chip captured the ultrastructure of HEVs, FRCs, and ECM fibrils in both paracortical and follicular compartments (Fig. 1F and **Fig. S1A**), closely resembling native lymphoid tissue morphology. Time-lapse imaging and 3D confocal reconstruction showed progressive self-assembly of vascular/reticular stromal networks and concomitant ECM deposition between day 1 and day 5 post-seeding (**Fig. S1B-D**). Consistent with chemokine guidance of lymphocyte positioning, CCL19 was detected along PDPN^+^ FRC tracks adjacent to CD3^+^ T cells (**Fig. S2A-B**). Notably, classical PDPN^+^ FRCs and CD157^+^ perivascular FRCs localized to distinct stromal zones adjacent to CD31^+^ endothelial cells (**Fig. S2C,D**), reflecting the spatial heterogeneity of lymph node stromal subsets observed in vivo. Functionally, co-culture with FRCs enhanced the secretion of IL-2, IL-21, and CXCL13 and increased CD69 expression on lymphocytes (**Fig. S2E-F**), confirming that stromal networks are indispensable for sustaining immune activation. In the B cell follicles, FDCs formed a supportive niche that promoted B cell clustering and follicular organization, functionally mimicking the role of FDCs in vivo (Extended Data Fig. 1F) (23). Immunofluorescence imaging showed CD138^+^ plasma cells and CD27^+^ memory B cells within the follicles, indicating functional B cell maturation (Fig. 1D). Notably, immunofluorescence imaging revealed the ex vivo formation of GC-like structures 10–12 days post-vaccination, characterized by dense clusters of CD19^+^Ki67^+^ proliferating B cells surrounded by IgD^+^ naïve and memory B cells (Fig. 1G) (8). We further demonstrated zonal organization of CD83^+^ GC B cells in light zone and CXCR4^+^ cells in dark zone of the GC structure. Together, these structural datasets indicate that the engineered chip recapitulates the stromal blueprint of the human lymph node.

We further applied scRNA-seq analysis to validate the biomimicry of the engineered lymph node niche to its in vivo counterpart. The scRNA-seq results validated that our lymph node chip maintained an enriched cellularity with immune and stromal cells, including DCs, monocytes, CD4^+^ and CD8^+^ T cells, regulatory T (T_reg_) cells, T follicular helper (T_fh_) cells, and naive and memory B cells, plasmablasts, fibroblasts and endothelial cells, comparable to that of the *in vivo* lymph node microenvironment (Fig. 1H). Notably, the LN-on-a-chip generated T_fh_ cells and plasmablast clusters, which are also present in primary human lymph node tissue but were absent in PBMCs. In contrast, fewer T_fh_ cells, memory B cells, and plasma cells that are associated with vaccine immunogenicity were detected in the unvaccinated group, whereas human PBMCs lacked stromal and follicular B cell populations. Upon vaccination (Fig. 1I), we observed elevated expression of functional immune markers such as B cell identity (CD27, CD38, MZB1) and T cell activation (CD3D, CD4, CD69). In parallel, we observed elevated expression of antigen presentation molecules (HLA-DRA), and vascular markers (PECAM1), suggesting coordinated enhancement of lymphocyte activation, GC maturation, and stromal–vascular crosstalk. These coordinated shifts, from stromal and metabolic modules toward lymphocyte activation and differentiation, underscore the chip’s capacity to recapitulate the early molecular hallmarks of GC-like responses upon antigen exposure.

### Vaccine induces DC maturation, T cell activation and intranodal migration in paracortex

**Fig. 2.**
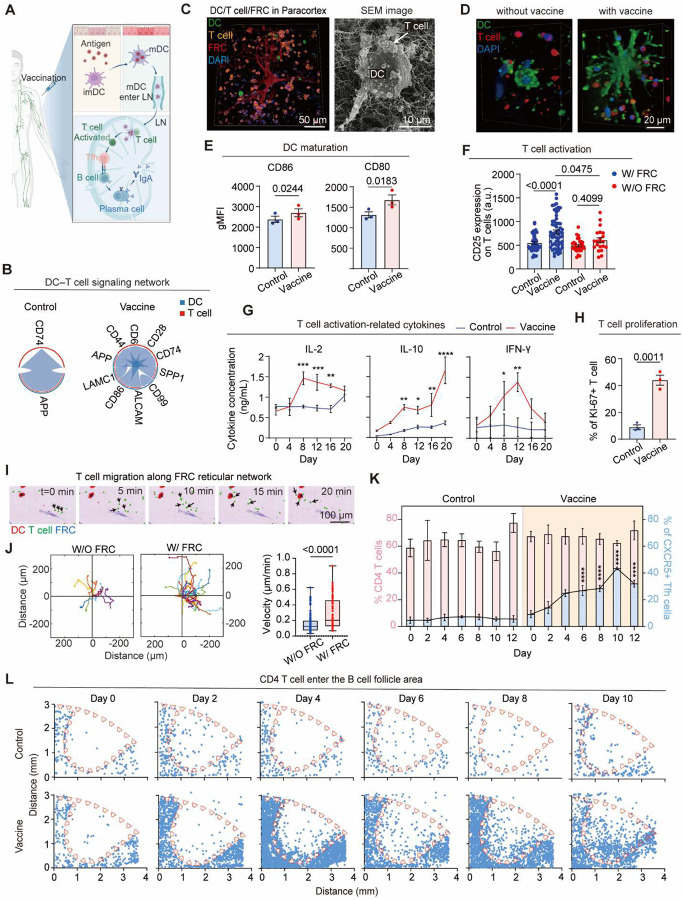
Vaccine-induced DC–T cell interactions within the LN-on-a-chip. (**A**) Schematic of vaccine-induced immune response in lymph node. (**B**) Inferring DC-T cell communication networks using CellChat from scRNA-seq data on day 10. (**C**) Confocal and SEM images showing DC, T cell and PDPN^+^ FRC in the paracortex. (**D**) Confocal images showing CD3^+^ T cells engaging CD11c^+^ DCs. (**E**) Quantification of DC maturation markers (CD80, CD86) on day 3 with flow cytometry (n = 3). (**F**) Quantification of T cell activation markers (CD25, n = 3 chips), (**G**) Temporal secretion profiles of T cell activation-related cytokines by ELISA (n = 3 independent experiments) in 20 days after vaccination. (**H**) Quantification of Ki67^+^ proliferating T cells on day 10 (n = 3 chips). (**I**) Time-lapse images showing T cell migration on FRC network and interaction with DC on day 3 post-vaccination. (**J**) Single-cell migration trajectories (left) and quantification of T cell motility (right) with or without FRC (>100 tracks per group; two biological replicates). (**K**) Ratios of CD4^+^ T cells and CXCR5^+^ T_fh_ cells in total T cells over 12 days post-vaccination (n = 3 technical replicates). (**L**) CD4^+^ T cell migration into the follicle region over 10 days post-vaccination.

To functionally validate the LN-on-a-chip model for simulating vaccine-induced adaptive immune responses, we introduced an influenza vaccine and longitudinally monitored resulting immunogenicity responses on chip (Fig. 2A). After vaccination, DCs capture and process vaccine antigens, then activate naïve T cells in the paracortex (24). To understand how vaccine stimulation reshapes the immune communication landscape within the paracortex niche, we first analyzed ligand-receptor (LR) interactions across the DC–T cell axis 10 days post-vaccination. Control chips without vaccination exhibited a highly restricted signaling network dominated by the APP–CD74 pair, reflecting tonic signaling under steady-state conditions. In contrast, vaccination induced a robust expansion of this network, incorporating multiple co-stimulatory and adhesion interactions, including CD86-CD28, ALCAM-CD6, and CD44-CD74, consistent with active T cell priming and immunological synapse formation (Fig. 2B).

Beside the immune cell interactions, the paracortex stromal niche also supports T cell activation and localization on chip. Immunofluorescence and SEM imaging confirmed the formation of tight DC-T cell-FRC clusters within the paracortex, where DCs extended dendrites to engage neighboring T cells along the reticular scaffold (Fig. 2C, D). Our results confirmed vaccine markedly promoted immature DCs (imDCs) transition into mature DCs (mDCs), as evidenced by up-regulated CD86 and CD80 expression (Fig. 2E, **Fig. S3A**), and led to robust T cell activation with elevated CD25 expression on CD4^+^ T cells in 10 days, an effect dependent on FRC support (Fig. 2F). Vaccination increased secretion of T cell activation related cytokines IL-2, IL-10, and IFN-γ (Fig. 2G), and a significant increase in Ki67^+^ proliferating T cells compared to compared to unvaccinated controls (Fig. 2H). Moreover, time-lapse imaging showed that T cells exhibited higher motility and directional velocity guided along the FRC reticulum (Fig. 2I) when interacting with vaccine group compared to unvaccinated (Fig. 2J), reflecting active scanning behavior during antigen surveillance (25–27). Notably, CD4^+^ T cells progressively upregulated CXCR5 and acquired a T_fh_ phenotype after vaccine stimulation (Fig. 2K). These activated T cells then migrate into the B cell follicle regions (Fig. 2L) and interact with B cells which are critical steps for downstream GC formation and B cell help (38–40).

### Vaccine induces B cell subset remodeling, GC formation and antibody production in the follicles

**Fig. 3.**
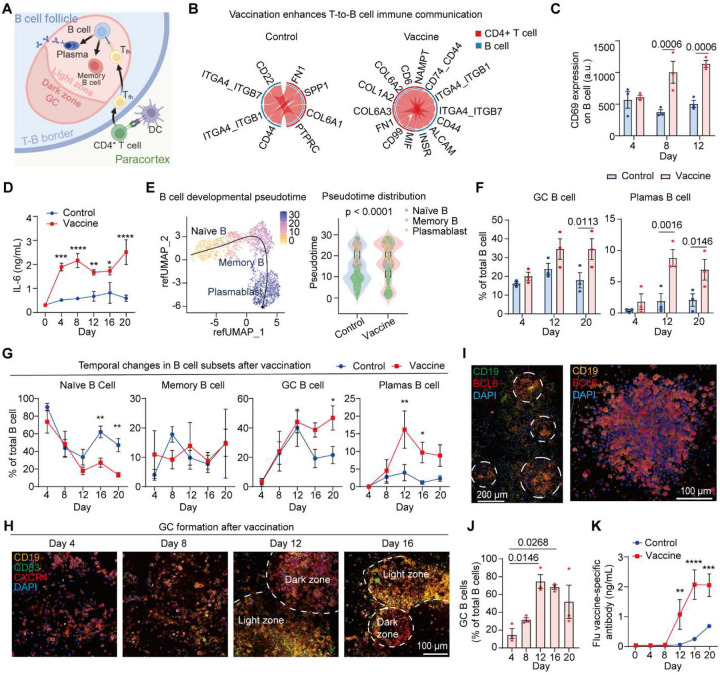
Vaccine-induced B cell activation, GC formation, and antibody secretion on chip. (**A**) Schematic showing T-B cell interactions across the paracortex-follicle interface. (**B**) Inferring T-B cell communication networks (interactions with P < 0.05, mean communication probability > 0.35) using CellChat from scRNA-seq data on day 10. (**C**) Time-dependent B cell activation (CD69) and (**D**) IL-6 secretion (ELISA) in control and vaccinated LN-on-a-chip cultures (n = 3 independent experiments). (**E**) Pseudotime trajectory of B cell maturation post-vaccination. UMAP visualization of B cells (Day 10) with overlaid Slingshot pseudotime (left). Violin plots (right) comparing pseudotime distributions between vaccine and control conditions with two-sided Wilcoxon rank-sum test. (**F**) Time-course analysis of B cell subsets showing population shifts in control and vaccinated LN-on-a-chip cultures by flow cytometry (n = 3 independent experiments) and (**G**) immunostaining (n = 4 independent experiments). (**H**) Immunofluorescence images showing GC formation and zonal organization at day 4–16 post-vaccination, with CD19^+^ B cells, CD83^+^ GC B cells, CXCR4^+^ dark-zone–associated cells, and nuclei (DAPI). (**I**) Confocal immunofluorescence image showing GC-like structures composed of CD19^+^ B cells with high BCL6 expression at day 12. (**J**) Percentage of CD19^+^BCL6^+^ GC B cells among total CD19^+^ B cells over time in vaccinated LN-on-a-chip cultures (n = 3 independent experiments). (**K**) Influenza-specific IgG levels in chip effluents measured by ELISA over 20 days (n = 3 independent experiments).

Upon vaccination, naïve B cells, with the assistance of T_fh_ cells, differentiate into different subtypes including plasma cells capable of producing antigen-specific antibodies (Fig. 3A) (28). To dissect how vaccination reshapes T-B intercellular communication in the follicles, we analyzed LR signaling between CD4^+^ T cells and B cells from the chip-retrieved cells 10 days after vaccination. In control chips without vaccine, T-B cell communication was minimal and centered largely on integrin-mediated adhesion and matrix interactions (e.g., ITGA4-ITGB1, ITGA4-ITGB7, FN1, COL4A1), reflecting a baseline, non-activated state (Fig. 3B). Vaccination markedly broadened this network, introducing additional inflammatory, costimulatory, and CD74-associated interactions (e.g., MIF, NAMPT, CD44, ALCAM, CD74), indicating that activated CD4^+^ T cells engage in more complex and directional crosstalk with B cells during early humoral priming. We further extended these observations from T–B interactions to all immune cell compartments with a CellChat-based network analysis. Representative chord diagrams highlighted strengthened signaling through chemokine, adhesion, and costimulatory pairs (including CCL3-CCR1, SPP1-CD44, CD6-ALCAM, and FN1-ITGA4/ITGB1), linking DCs, CD4^+^ T cells, and B cells more robustly in vaccinated chips relative to controls (**Fig. S4A**). At the network level, vaccination markedly increased global interaction strength across immune and stromal compartments (**Fig. S4B**). Notably, the connection emerged between lymphocytes and fibroblasts, monocytes, and endothelial cells, indicating coordinated remodeling of antigen presentation and stromal support pathways that facilitate immune cell recruitment and activation. To connect these cellular responses with underlying transcriptional programs, we performed Gene Ontology enrichment analysis on vaccine-induced genes. GO (Biological Process) terms showed strong enrichment of immune-activation pathways in vaccinated chips (**Fig. S4C**), including αβ T cell activation, TCR and antigen-receptor (AgR) signaling, immune-receptor signaling, T cell differentiation, and lymphocyte proliferation. These signatures indicate robust engagement of antigen-driven T cell programs and enhanced intercellular communication within the chip following vaccination.

We next examined whether vaccine-induced activation translated into functional B cell differentiation by first assessing early activation and cytokine outputs, followed by transcriptomic trajectory analysis. Vaccinated LN-on-a-chip cultures exhibited a significantly higher proportion of activated CD69^+^ B cells compared with unstimulated controls (Fig. 3C), accompanied by a progressive increase in IL-6 secretion over time (Fig. 3D), consistent with the establishment of a cytokine milieu permissive for B cell activation and differentiation. To assess global changes in B cell developmental state, we performed pseudotime trajectory analysis on scRNA-seq data (Fig. 3E). In vaccinated chips, B cells progressed along a continuous trajectory from naïve to memory and plasmablast states, resulting in a significantly extended pseudotime distribution compared with controls 10 days after vaccination. In contrast, B cells from control chips remained enriched at early pseudotime positions, indicating limited activation and minimal transition into effector states. Together, these results demonstrate that vaccination reprograms the B cell developmental landscape on chip, shifting it from a static, naïve-dominated state toward a dynamic, antigen-driven trajectory culminating in plasmablast differentiation. To capture the temporal evolution of the B cell response, we performed longitudinal phenotyping of B cells over 20 days using flow cytometric analysis (Fig. 3F and **Fig. S3D**) and immunostaining of CD27 and CD138 (naïve: CD27^−^CD38^−^, GC: CD27^+^CD38^+^, memory: CD27^+^CD38^−^, and plasma: CD27^+^CD38^++^, **Fig. S5**) to classify B cell states (9). Vaccination induced a clear progression from naïve to GC phenotypes, followed by the emergence of plasma B cells at later time points (Fig. 3F, G), well mirrored canonical GC fate trajectories observed in vivo (29, 30).

**Extended Data Fig. 2.**
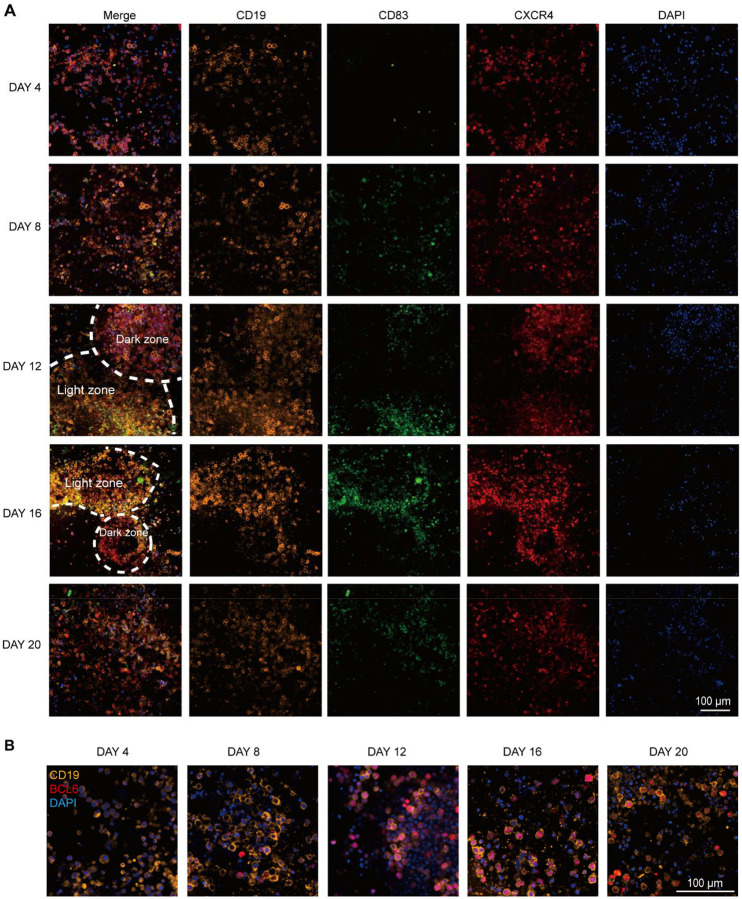
Temporal dynamics of GC formation and zonation in the LN-on-a-chip model. (**A**) Immunofluorescence images showing B cell follicular region of the lymph node chip at days 4, 8, 12, 16, and 20 post-vaccination, revealing distinct GC-like structures formed over time. B cells were identified by CD19 and nuclei (DAPI), proliferative GC B cells by CD83, and dark zone–associated cells by CXCR4. Dashed lines indicate boundaries of light zone and dark zone. (**B**) Representative immunofluorescence images showing CD19, BCL6, and nuclei (DAPI) staining at indicated time points. CD19^+^BCL6^+^ cells were identified as GC B cells.

Our human lymph node model recapitulated the spontaneous formation of organized GC structures in the B follicle regions, a hallmark of efficient adaptive immune response following vaccination (Fig. 3H–J and Extended Data Fig. 2) (31). We examined the spatiotemporal organization of GC structures using CD19, CD83, and CXCR4 staining. Early post-vaccination (day 4–8), only scattered B cells were observed, without organized GC architecture or zonation. By day 12–16, multiple compact GC-like structures were observed throughout the LN-on-a-chip, displaying CXCR4^+^ dark zone and CD83^+^ light zone organization and enriched for CD19^+^BCL6^+^ B cells (Fig. 3H and Extended Data Fig. 2A). This is consistent with decreased B cell cycling and progression toward GC contraction (32). To verify molecular features of GC formation, we analyzed BCL6 (Fig. 3I), the canonical GC B cell regulator (33). Quantitative analysis revealed that the proportion of CD19^+^BCL6^+^ GC B cells among total B cells increased over time and reached its maximum at day 12 (Fig. 3J, Extended Data Fig. 2B). To assess functional output, we measured influenza-specific IgG in chip effluents over 20 days. Consistent with the GC formation and plasma B cell differentiation of on chip, a marked increase in influenza vaccine–specific antibody secretion was observed from day 8 onward in vaccinated chips, with antibody concentrations continuing to rise through day 20 (Fig. 3I). In contrast, control chips showed only minimal antibody production over the same period. Together, these results demonstrate that our LN-on-a-chip well recapitulated the vaccine immunogenicity events, showing coordinated lymphocyte activation, strengthened intercellular crosstalk, B cell differentiation, GC formation and influenza-specific antibody responses in vitro.

### Clinical-trial-on-chip study for a precise and individualized evaluation of vaccine responses

**Fig. 4.**
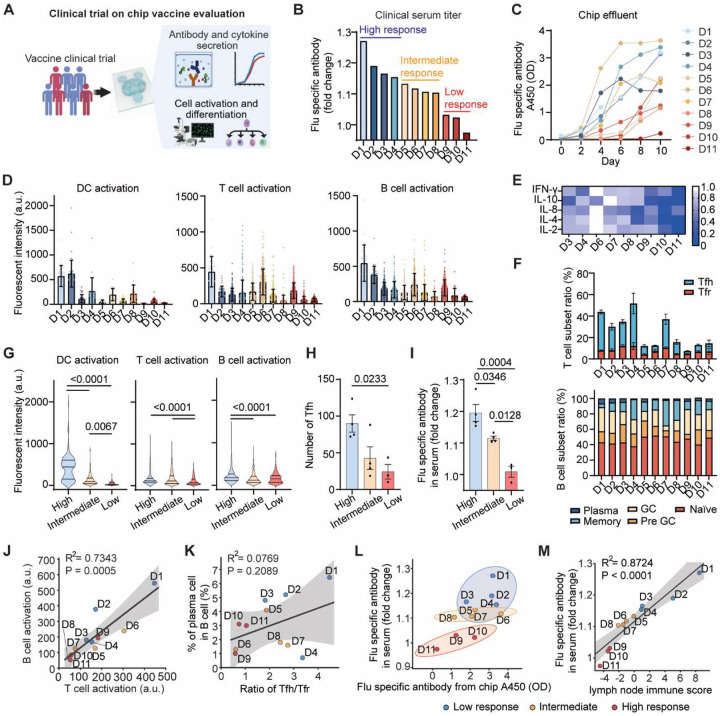
Clinical trial-on-a-chip evaluation of donor-specific variation in vaccine immunogenicity. (**A**) Schematic of clinical trial-on-a-chip study. (**B**) Fold-change in serum influenza-specific antibody one month after vaccination, measured by a multiplex influenza immunoassay. (**C**) Time-course influenza-specific IgG secretion measured by ELISA from chip (mean of three technical replicates). (**D**) Quantification of DC (CD86), T cell (CD25), B cell (CD69) activations on day 10 (n = 3 chips). (**E**) Heatmap of cytokine levels (log_2_ fold change) on day 10, measured by ELISA from effluent pooled from 6 replicating chips. (**F**) Ratio of T_fh_/T_fr_ cells (top) and B cell subtypes (bottom, mean of 3 chips) on day 10. (**G**) Comparison of immune cell activation levels, (**H**) ratio of T_fh_/T_fr_ cells, and (**I**) serum flu-specific antibody levels across high-, intermediate-, and low-response groups defined in B. (**J**) B cell activation level correlates with T cell activation level across donors. (**K**) Ratio of T_fh_/T_fr_ cells correlates with plasma cell output. (**L**) Chip-measured antibody secretion by ELISA mirrors serum IgG fold changes. Ellipses denote 90% confidence intervals. (**M**) Correlation of serum IgG fold change against lymph node immune score. Correlation analyses in panels J, K, and M were performed using Pearson correlation with linear regression. Gray shaded regions represent 95% confidence intervals.

In parallel with an influenza vaccine clinical trial, we established personalized LN-on-a-chip models using participant-matched PBMCs to conduct a clinical-trial-on-chip study of donor-specific vaccine responses (Fig. 4). The influenza vaccine clinical trial serum data revealed a broad spectrum of influenza-specific IgG production post-vaccination, due to factors of age, gender and comorbidities (Fig. 4B, **Table S1**). Donors were stratified into high, intermediate, and low responders based on their clinical serum IgG fold changes, providing a benchmark for interpreting on-chip immune readouts. Notably, influenza-specific antibody production from chip effluents overall mirrored the clinical hierarchy, with high-responder donors showing rapid and sustained antibody secretion, whereas low responders produced minimal titers throughout the 10-day period (Fig. 4C). Quantitative analyses confirmed substantial donor-to-donor variability in levels of DC (CD86), T cell (CD25), B cell (CD69) activation (Fig. 4D) and activation-related IFN-γ, IL-1β, IL-4, IL-8, and IL-2 cytokine secretions (Fig. 4E), as well as proportions of CD4^+^CXCR5^+^ T_fh_ to CD4^+^CXCR5^+^FOXP3^+^ T_fr_ cells in the paracortex and B cell subsets within the B cell follicle regions (Fig. 4F). Groupwise analysis results showed stronger DC, T and B cell activations, significantly higher T_fh_ counts and antibody production in high versus intermediate and low responders (Fig. 4G–I). Correlation analyses further demonstrated coordinated activation among immune compartments. T and B cell activation levels were strongly correlated across donors (Fig. 4J), and the plasma cell-to-T_fh_ ratio positively associated (Fig. 4K), underscoring the functional coupling between T cell help and B cell differentiation and humoral output.

**Extended Data Fig. 3.**
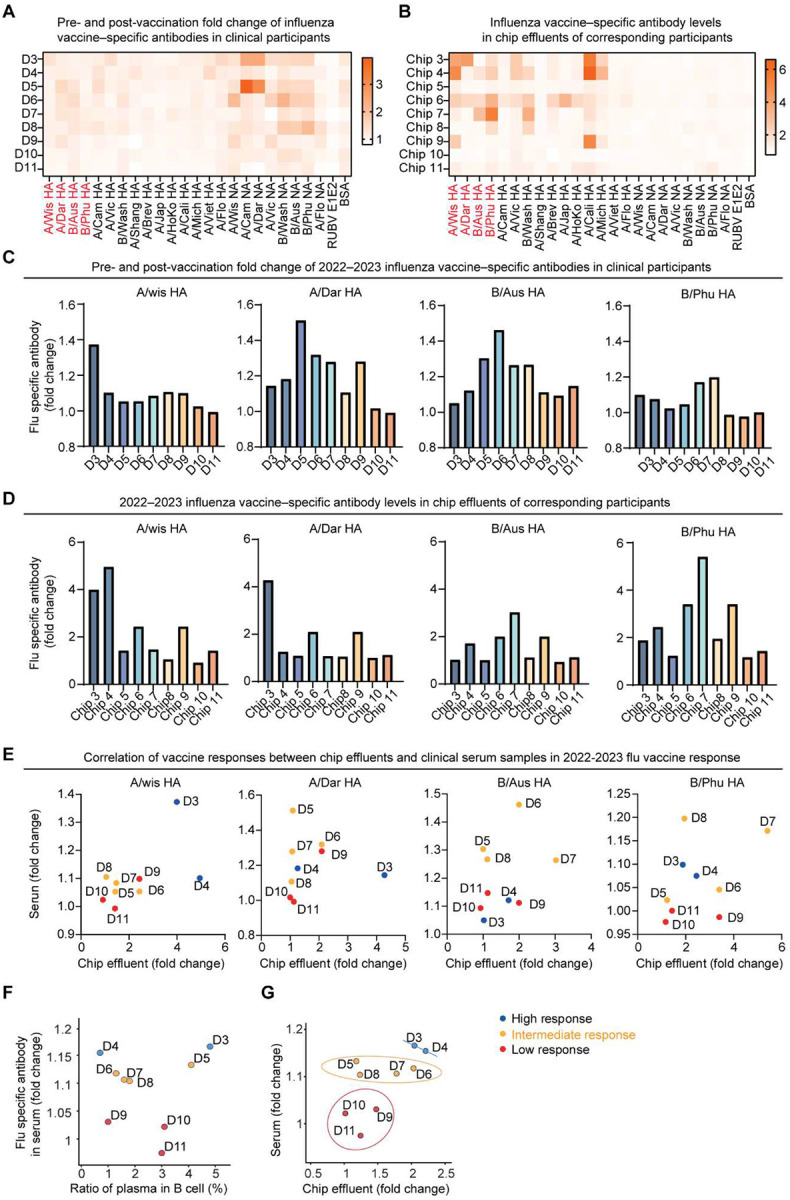
Chip-measured influenza vaccine–specific antibody responses correlate donors’ serum responses. (**A**) Heatmaps showing clinical-measured and (**B**) chip-measured post-vaccination IgG binding across 23 influenza A/B HA and NA antigens and rubella E1E2 protein in response to the 2022–2023 influenza vaccines in all clinical trial participants, measured by a bead-based multiplex influenza immunoassay. (**C**) Fold change in serum IgG binding post- versus pre-vaccination for four 2022–2023 seasonal influenza HA antigens (A/Wisconsin, A/Darwin, B/Austria, and B/Phuket), and (**D**) corresponding IgG levels in chip effluents from the same donor. (**E**) Correlation between chip-measured and clinical serum IgG responses for each of the four HA antigens. (**F**) Correlation between serum antibody response and ratio of plasma cells among CD19^+^ B cells on chip. (**G**) Correlation between chip-measured and clinical serum antibody responses. In G, ellipses represent 90% confidence intervals for different vaccine-response groups. For all serum antibody measurements, serum samples were collected before vaccination and again approximately one month after vaccination for antibody quantification, n = 1 per donor. For chip measurements, each data point represents the mean normalized value measured from a medium pooled from 6 replicate chips. Antibody binding intensities were background-subtracted and normalized using IC45 values. Data F and G represent the mean binding values averaged across the HA antigens.

We further profiled vaccine-specific IgG binding across donors using a 23-antigen multiplex influenza immunoassay encompassing historical and 2022–2023 HA and neuraminidase (NA) variants (Extended Data Fig. 3, **Table S3**). Clinical serum samples and corresponding chip effluents showed highly concordant binding spectra across the 23 antigens (Extended Data Fig. 3A,B). Particularly, the four main HA antigens contained in the 2022–2023 vaccine (A/Wisconsin, A/Darwin, B/Austria, and B/Phuket) parallelly increased both in serum and chip samples (Extended Data Fig. 3C–E). As shown in Extended Data Fig. 3F, the serum influenza-specific antibody fold changes strongly correlated with the proportion of plasma B cells on-chip. Importantly, the influenza-specific antibody levels in chip effluents both measured by ELISA (Fig. 4L) and a multiplex influenza immunoassay (mean IgG fold changes across 15 influenza hemagglutinin (HA) antigens Extended Data Fig. 3G) closely matched the clinically measured IgG fold changes in participant sera, and distinguished between the different clinical responder groups. Collectively, we integrated these key chip-measured immune metrics, including influenza-specific antibody secretion, DC, T, and B activation, T_fh_/T_fr_ ratio, and plasma cell and GC B cell proportions, into a composite lymph node immune score to define each donor’s immunogenicity capacity. The lymph node immune score shows a strong correlation with clinical serum responses (Fig. 4M), validating the predictive capacity of our chip model. Together, these data show that the individualized LN-on-a-chip captured donor-specific immune heterogeneity that quantitatively parallels clinical vaccine efficacy.

### Investigating the impact of age, gender, and comorbidities on vaccination efficacy.

**Extended Data Fig. 4.**
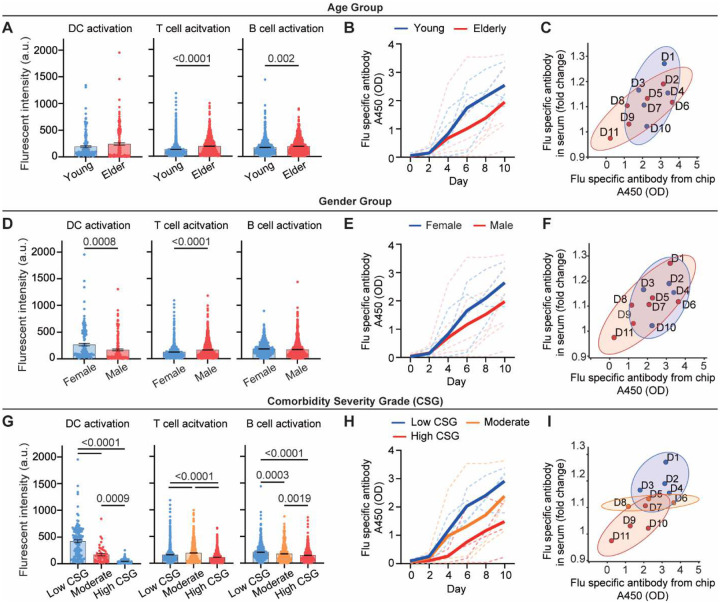
Stratification of donor-specific vaccine responses by age, gender, and comorbidity severity. (**A**) Comparison of DC (CD86), T cell (CD25), and B cell (CD69) activation levels, (**B**) influenza-specific antibody secretion in chip effluents between young (<65 y, n = 5) and elderly (≥65 y, n = 6) donors. (**C**) Correlation between antibody secretion in chip effluents and clinical serum titers between age groups. (**D**) Comparisons of DC, T cell, and B cell activation levels, (**E**) influenza-specific antibody secretion in chip effluents between genders (female n = 4, male n = 7). (**F**) Correlation between antibody secretion in chip effluents and clinical serum titers across donors of different genders. (**G**) Comparison of DC, T cell, and B cell activation levels, (**H**) influenza-specific antibody secretion in chip effluents between across groups stratified by comorbidity severity grade (CSG, defined in Table 1; High CSG n = 4, moderate CSG n = 3, and low CSG n = 4). (**I**) Correlation between antibody secretion in chip effluents and clinical serum titers across CSG groups. Clinical serum antibody titers were measured using a bead-based multiplex immunoassay, antibody in chip effluents was measured by ELISA. Data in A, C, D, F, G, and I were measured on day 10 after vaccination. In B, E, H, thick lines represent the mean of the group, dash lines represent individual donors. In C, F, I, ellipses represent 90% confidence intervals for different groups.

**Fig. 5.**
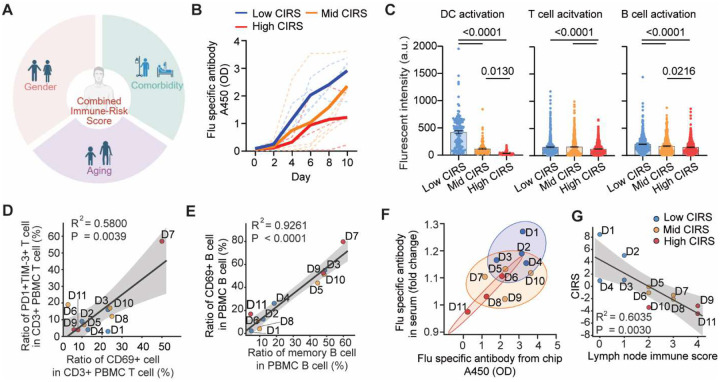
Vaccine response variance influenced by age and comorbidity immune risk. (**A**) Stratification of trial participant vaccine responses by composite immuno-risk score (CIRS), combining age and comorbidity. (**B**) Influenza-specific antibody production stratified by CIRS. Thick lines represent the mean of the group, dash lines represent individual donors. (**C**) Quantified DC, T, and B cell activation levels in chips stratified by CIRS (high CIRS n = 9 chips from 3 donors, Mid CIRS n = 12 chips from 4 donors, low CIRS n = 12 chips from 4 donors; each dot represents one cell). (**D**) Correlation between the ratios of exhausted PD-1^+^TIM-3^+^ T cells and basal activated CD69^+^ T cells within donors’ CD3^+^ PBMC T cells before vaccination. (**E**) Correlation between CD69^+^ B cells and memory B cells in pre-vaccination PBMC B cells. Data in D, E were obtained from a single flow cytometry experiment on trial participants’ PBMCs. (**F**) Correlation between flu-specific antibody production from the LN-on-a-chip and matched clinical serum IgG titers across donors. Ellipses represent 90% confidence intervals. (**G**) Negative correlation between the CIRS and the lymph node immune score. Correlation analyses in panels D, E, and G were performed using Pearson correlation with linear regression. Gray shaded regions represent 95% confidence intervals.

In the clinical-trial-on-chip study, we investigated the impact of age, gender, and comorbidities on the heterogeneity in donors’ vaccine immunogenicity (Fig. 5A, **Table S1**). When donors were grouped by age, younger participants (< 65 y) exhibited markedly stronger immune activation and antibody production than elderly donors (≥ 65 y) (Extended Data Fig. 4A–C). We examined potential gender-associated variation in immune activation. Overall, female donors displayed modestly stronger activation of DCs, whereas male donors displayed higher T cell activation (Extended Data Fig. 4D). These cellular differences were accompanied by only minor sex-related variation in influenza-specific antibody production both in chip effluents and corresponding clinical sera (Extended Data Fig. 4E,F), consistent with the moderate effect size of gender on vaccine responsiveness observed clinically. Finally, we analyzed the impact of comorbidity severity on vaccine-induced immune performance. A comorbidity severity grade (CSG; 0–3) was determined for each donor according to the type and severity of chronic conditions (**Table S1**). Donors with low CSG (no or mild chronic conditions) exhibited strong DC, T, and B cell activation and influenza-specific antibody secretion, whereas those with moderate or high CSG showed progressive delayed and attenuated responses (Extended Data Fig. 4G,H). While the age- and gender-dependent clustering of donors were less distinct in the correlation plots (Extended Data Fig. 4C,F), correlation between chip-derived and clinical serum antibody titers (Extended Data Fig. 4I) confirmed that the LN-on-a-chip reliably captured comorbidity-associated variability. Overall, a higher CSG corresponds to weaker humoral output, which aligns with clinical evidence linking chronic inflammatory and metabolic conditions to impaired vaccine-induced humoral immunity.

We defined a composite immuno-risk score (CIRS), categorizing donors into low-, mid-, and high-risk groups that reflect cumulative age- and disease-associated immune burden (**Table S1**). Across CIRS strata, on-chip influenza-specific antibody production recapitulated the expected risk gradient. Low-CIRS donors exhibited rapid and sustained IgG increases over 10 days, mid-CIRS donors showed intermediate kinetics, and high-CIRS donors displayed markedly blunted responses (Fig. 5B). Cellular readouts mirrored these functional outcomes: quantitative imaging revealed progressive reductions in DC (CD86), T cell (CD25), and B cell (CD69) activation with increasing CIRS (Fig. 5C), indicating that cumulative immune risk is associated with weaker vaccine-elicited activation on chip. To understand how these functional differences relate to each donor’s intrinsic immune baseline, we profiled pre-vaccination PBMCs’ exhaustion and basal immune activation signatures (Fig. 5D,E). Results shows that donors with elevated basal CD69^+^ T cell activation also carried higher PD-1^+^TIM-3^+^ exhaustion signatures (Fig. 5D). Likewise, memory B cell abundance correlated with CD69^+^ B cell activation in donor’s PBMC (Fig. 5E), suggesting that donors with expanded memory pools maintain higher basal activation. Notably, the HIV^+^ high-CIRS donor (D7) exhibited the strongest co-expression pattern, consistent with chronic immune stimulation and reduced vaccine responsiveness associated with HIV infection. These pre-vaccination immune cell profiles reveal a donor-intrinsic immune heterogeneity: high-CIRS donors displayed a hyperactivated yet exhausted phenotype, whereas low-CIRS donors maintained a more balanced, resting state. Consistent with this stratification, influenza-specific IgG levels produced on-chip strongly correlated with matched serum titers measured from the clinical trial (Fig. 5F), and the composite lymph node immune score revealed a significant negative correlation with CIRS (Fig. 5G), confirming that increased age- and comorbidity-associated immune risk was quantitatively linked to impaired adaptive immune responses.

### scRNA-seq analysis reveals key DC–T_fh_–B cell signaling network shaping vaccine immunogenicity

**Fig 6.**
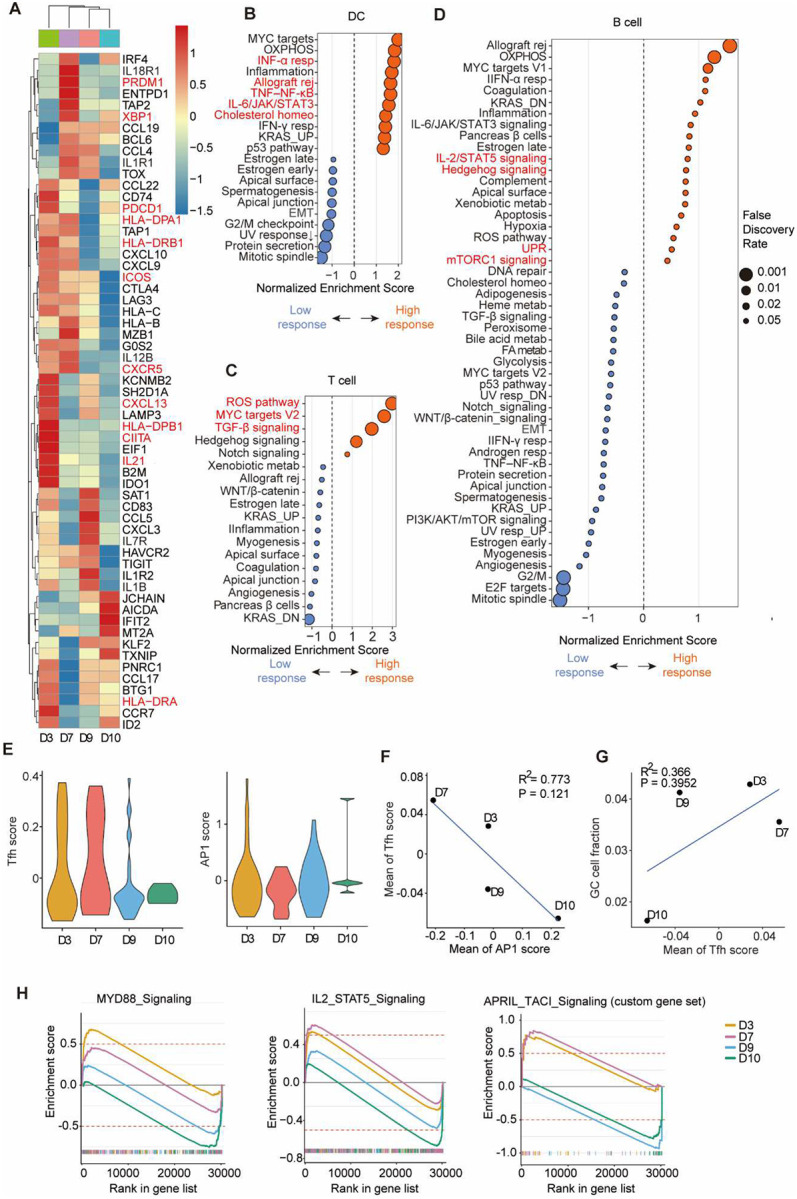
scRNA-seq analysis of differences in immune pathway between high-, intermediate- and low-response donors. (**A**) Heatmap of selected immune-related genes across donor hashtags, shown as row-centered z-scored log-CPM pseudobulk expression (per donor × cell type), clustered by correlation (complete linkage) and capped at ±3. High (D3) and intermediate (D7) responders displayed upregulation of inflammatory and antigen-presentation modules, including TLR/MyD88-driven IFN-α/β, TNF–NF-κB, and IL-6–STAT3 signaling together with enhanced MHC I/II expression, whereas low responders (D9, D10) preferentially engaged stress- and metabolic-associated signatures. (**B–D**) Hallmark GSEA was performed separately for DCs (B), T cells (C), and B cells (D). Genes were ranked using Seurat FindMarkers (Wilcoxon rank-sum test comparing high vs low responders; no logFC or percentage filters applied; ranking metric = avg_log2FC). Enrichment was tested with fgsea against the MSigDB Hallmark collection (minSize=10, maxSize=500, nperm=10,000). The plots show normalized enrichment score (NES) on the x-axis, enrichment direction (color: orange for high, blue for low responders), and false discovery rate (FDR, Benjamini–Hochberg) represented by point size. (**E**) Violin plots showing single-cell UCell enrichment scores for the curated T_fh_ gene signature (BCL6, CXCR5, ICOS, PDCD1, MAF, SH2D1A, TOX2; left) and AP-1 signature (FOS, FOSB, JUN, JUNB, JUND, ATF3, BATF; right) across donors (D3, D7, D9, D10). Distributions illustrate donor-specific heterogeneity and partially overlapping score ranges. No statistical comparisons were performed at the donor level for these distributions. (**F**) Donor-level correlation between AP-1 activity and T_fh_ program strength. Each dot represents one donor (n = 4). Linear regression analysis is shown with coefficient of determination (R^2^) and P value indicated in the plot. (**G**) Donor-level association between T_fh_ program strength and GC fraction. GC fraction was quantified using UCell enrichment of a curated GC B cell gene signature (BCL6, AICDA, S1PR2, RGS13, MEF2B, CXCR4). Linear regression analysis is shown with R^2^ and P value indicated in the plot. (**H**) GSEA plots of hallmark immune signaling pathways highlight donor-dependent differences in immune cell activation.

To mechanistically understand the inter-donor immune heterogeneity, we performed scRNA-seq analysis on cells 10 days post-vaccination recovered from chips of four representative clinical trial participates, including one high (D3) and one intermediate (D7) vaccine responders, and two low responders (D9, D10) (Fig. 6). Unsupervised clustering identified 13 cell populations encompassing DCs, CD4^+^/CD8^+^ T cells, and B/plasma cells (**Fig. S6A**), consistent with the lineage distribution in our reference atlas (Fig. 1H). Differential gene-expression analysis of immune cells (DCs, T cells, and B cells) revealed that high responders exhibited a globally more coordinated DC–T_fh_–B cell immune axis than intermediate and low responders (Fig. 6A). At the DC level, high responder (D3) showed strong up-regulation of antigen-presentation and co-stimulatory machinery genes (HLA-DPA1, HLA-DPB1, HLA-DRA, HLA-DRB1, CIITA), suggesting efficient antigen processing and T cell priming. Moreover, high responder displayed marked induction of T_fh_ programs characterized by CXCR5, PDCD1 (PD-1), ICOS, and IL21, indicating enhanced follicular homing, activation, and B cell help. Elevated CXCL13 further supported the establishment of an organized follicular microenvironment that promotes productive T–B cell communication. Moreover, intermediate responder (D7) showed increases in genes involved in GC induction (BCL6), class-switch recombination and plasma cell differentiation (XBP1, PRDM1).

Low responders (D9, D10) lack of key T_fh_–GC related genes such as BCL6, CXCR5, CXCL13, and ICOS, but showed strong upregulation of interferon- and stress-response genes (IFIT2, MT2A, TXNIP) together with persistently high KLF2, a transcription factor that restrains T_fh_ differentiation (34). This transcriptional profile suggests that, while humoral programs may be initiated as indicated by detectable expression of AICDA and JCHAIN, they fail to progress in a coordinated manner across DC, T, and B cell compartments, resulting in an impaired DC–T_fh_–B cell axis and suboptimal antibody production. Consistent with this observation, the volcano plot comparison (**Fig. S6B**) demonstrated a clear transcriptional polarization between high/intermediate- and low-responder groups. High/intermediate responders were enriched with genes related to antigen presentation (HLA-C, B2M) and metabolic activation (EEF1A1, RPL9, RPS18), whereas low responders expressed higher levels of inhibitory and regulatory transcripts (FCRL5, NEAT1, SNHG5, ERAP2), suggesting an immunoregulatory or hyporesponsive state. Together, these results indicate that high/intermediate responders sustain a mature, antigen-presenting, and metabolically active immune network, whereas low responders exhibit features of impaired DC maturation, reduced T_fh_ differentiation, and suboptimal B cell activation.

To determine which transcriptional pathways distinguish high and low vaccine responders, we performed Hallmark GSEA separately for DCs, T cells, and B cells (Fig. 6B–D). Across all three immune cell population, high/intermediate responders (D3, D7) consistently exhibited enrichment of pathways that are canonically engaged during vaccine-induced adaptive immune activation. In DCs (Fig. 6B), high-response group showed strong positive enrichment of Interferon-α response (INF-α resp), TNF-α/NF-κB signaling, IL-6/STAT3 signaling, cholesterol homeostasis, and allograft rejection. These pathways reflect a more robust innate cytokine and inflammatory activation state, which is typically required for efficient antigen sensing and priming after vaccination. In T cells (Fig. 6C), the high-response group upregulated ROS pathway, MYC targets V2, and TGF-β signaling, indicating a transcriptional profile consistent with enhanced T cell activation and expansion during the early adaptive phase of the vaccine response. Importantly, the consistent upregulation of MYC target gene sets across immune compartments highlights a shared transcriptional program associated with stronger vaccine responsiveness. In B cells (Fig. 6D), high-response group displayed stronger enrichment of IL-2/STAT5 signaling, hedgehog signaling, unfolded protein response (UPR), and mTORC1 signaling pathways associated with B cell activation, metabolic upregulation, and plasmablast differentiation that support antibody generation following vaccination. Notably, although MYC target pathways were increased in high responder, classical proliferation-associated gene sets such as E2F targets and G2/M checkpoint showed limited enrichment on day 10. It is consistent with the well-established kinetics of human vaccine responses that the B cell shift from early proliferative responses around days 3–7 toward differentiation by day 7–14 (35).

Aging and comorbidities may result in chronic inflammation which diminish in donors’ vaccine immunogenicity. To further assess whether inflammatory transcriptional programs were associated with T_fh_ and GC capacity, we quantified donor-level T_fh_ and AP-1 signature scores using UCell enrichment. The T_fh_ score was calculated from a curated T_fh_ gene set (BCL6, CXCR5, ICOS, PDCD1, MAF, SH2D1A, TOX2), and the AP-1 score from AP-1–associated transcription factors (FOS, FOSB, JUN, JUNB, JUND, ATF3, BATF). T_fh_ scores displayed heterogeneous and partially overlapping distributions across donors, with D3 and D7 showing a greater proportion of higher-scoring cells, D9 and D10 exhibiting fewer high-scoring cells (Fig. 6E). AP-1 signature scores likewise varied across donors, with D3 and D9 containing more cells with elevated AP-1 scores, whereas D7 and D10 showed predominantly low-scoring cells. At the donor level, AP-1 activity showed an inverse correlation with T_fh_ program strength (Fig. 6F). T_fh_ scores showed a positive association with GC fraction—quantified using a curated GC B cell gene signature (BCL6, AICDA, S1PR2, RGS13, MEF2B, CXCR4, Fig. 6G). Together, these analyses suggest that variability in inflammatory transcriptional programs may be associated with differences in T_fh_-linked GC responses across donors.

We next performed pathway-level enrichment analysis for each donor across major immune lineages (Fig. 6H). In this analysis, MYD88 and IL-2/STAT5 signaling were evaluated using MSigDB Hallmark gene sets, whereas APRIL–TACI signaling was assessed using a custom gene set reflecting the BAFF/APRIL–TACI/BCMA axis (see Supplementary Materials). This analysis revealed that high (D3) and intermediate (D7) responders comparing to low responders (D9, D10) displayed coordinated enrichment of three key signaling checkpoints MYD88, IL-2/STAT5, and TACI that collectively shape the efficiency of the DC–T–B immune cascade on-chip. DC-intrinsic MyD88 signaling is indispensable for linking innate sensing to effective T cell priming and antibody production (36, 37). Enrichment of MyD88 signaling followed a clear gradient, reflecting distinct downstream T/B cell responses among high, intermediate and low responders (Fig. 6H). It was consistent with a transition from early TLR-driven sensing to a more mature antigen-presenting phenotype dominated by NF-κB/IRF3/7 and IL-6–STAT3 modules. At the T cell level, variability centered on the IL-2/STAT5 pathway, which governs T cell proliferation and helper potential (38–40). High/intermediate responders (D3, D7) exhibited relatively stronger IL-2/STAT5 pathway activity than the low responders (D9, D10), reflecting distinct IL-2 responsiveness among different responders. At the B cell level, we constructed a TACI/BCMA-associated plasma cell–survival pathway module based on the BAFF/APRIL–TACI/BCMA–PI3K–AKT–NF-κB–MCL1 signaling axis in human B cells (41). The TACI/BCMA pathway clearly stratified the high/intermediate and low responders. Studies show that BCMA deficiency leads to loss of long-lived plasma cells and reduced vaccine-specific antibody titers (42). In vaccine contexts, APRIL or BAFF molecular adjuvants have been shown to expand antibody-secreting B cell pools and improve serum responses (43, 44).

To validate these transcriptional findings at the level of cell-cell communication, we next analyzed donor-specific LR interaction networks using paired RNA and surface-protein data (TotalSeq-A) (**Fig. S6C–F**). High/intermediate-responder samples (D3, D7) showed enriched activation and “helping” interactions—ICOS–ICOSL, CD40–CD40LG, CXCL13–CXCR5, and IL-6–IL-6R—reflecting strong coordination across the DC–T_fh_–B cell network. In contrast, low responders (D9, D10) displayed relatively more inhibitory or regulatory interactions such as CTLA4–CD86 and TGFB–TGFBR, consistent with a restrained effector program and a more suppressive immune microenvironment. Together, these integrated single-cell and interaction analyses delineate mechanistic bottlenecks in low responders, from inefficient DC maturation to T_fh_ suppression and limited plasma-cell durability that serve as actionable targets for rational immune-enhancement strategies explored in subsequent experiments.

### Augmentation of DC–T–B cell signaling enhances vaccine immunogenicity on chip

**Extended Data Fig. 5.**
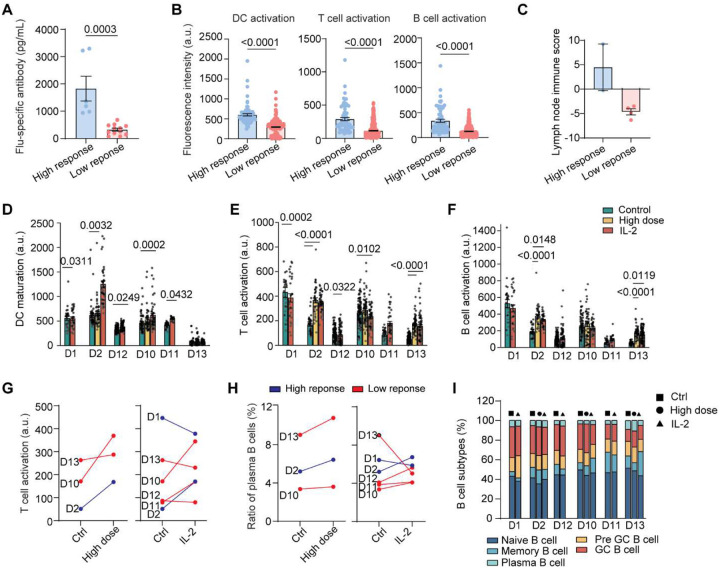
On-chip analyses of donor-specific vaccine responses to different immunomodulatory strategies. (**A**) Comparison of influenza-specific IgG secretion measured by ELISA from chip effluents (high responders: n = 6 replicates from 2 donors; low responders: n = 12 replicates from 4 donors), (**B**) DC (CD86), T cell (CD25), and B cell (CD69) activation levels (3 technical replicate chips per condition), and (**C**) the composite lymph node immune score indicating between high- (n = 2 donors) and low-response (n = 4 donors) groups. (**D**-**F**) Quantification of DC, T cell, and B cell activation levels across donors and immunomodulation conditions (n = 3 chips per condition). (**G**) T cell activation responses, (**H**) ratio of plasma B cells, and (**J**) ratio of B cell subtypes of individual donors under each immunomodulation condition. All measurements were performed on day 10 post-vaccination. Data point in G-I represents the mean of three replicate chips. Donor numbers for each condition were: High-dose n = 3, IL-2 n = 6, with matched Ctrl values from the same donors.

**Fig. 7.**
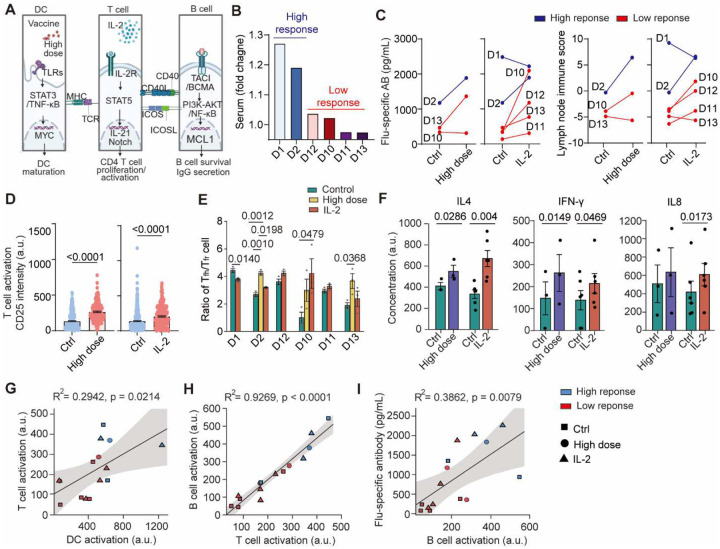
Augmentation of DC–T–B cell signaling enhances vaccine immunogenicity on chip. (**A**) Schematic of pathway-specific interventions targeting key immune signaling axes identified in transcriptomic analysis. (**B**) Fold change of influenza-specific antibody level in trail participants’ serums one month after vaccination (n=1 per donor), measured by a multiplex influenza immunoassay and stratified as high and low responses based on antibody secretion levels. (**C**) ELISA-measured influenza-specific antibody level (left) and lymph node immune score (right) from chips of high- and low-responder donors under normal vaccination control (Ctrl), high-dose vaccination, or vaccine^+^IL-2 conditions. Each data point represents the mean of three replicate chips on day 10 (n=3). (**D**) IL-4, IFN-γ, and IL-8 cytokine secretion levels measured by ELISA from effluents pooled from three replicate chips, (**E**) quantified T cell activation based on CD25 expression (High-dose n=3, IL-2 n=6 with matched Ctrl values), and (**F**) ratio of CD4^+^CXCR5^+^ T_fh_ cells to CD4^+^CXCR5^+^FOXP3^+^ T_fr_ cells across conditions on day 10. (**G**-**I**) Correlation analyses reveal hierarchical coupling among DC, T cell, and B cell responses on-chip. Each data point represents the mean of three chips; correlation analyses were performed using Pearson correlation with linear regression; gray shaded regions represent 95% confidence intervals.

The donor-resolved scRNA-seq analysis identified three mechanistic nodes MYD88, IL-2/STAT5, and TACI that collectively shape the efficiency of the vaccine immunogenicity. These axes represent potential targets of immunomodulatory interventions for enhancing vaccine responses (Fig. 7A): high-dose vaccination boosts MyD88-NF-κB/IRF3/7-dependent DC maturation for efficient priming; recombinant IL-2 cytokine adjuvant activates IL-2R–STAT5 to enhance T cell activation and proliferation thus to support B cell differentiation and antibody secretions. We thus performed a trial-on-chip study of these immunomodulatory interventions using our LN-on-a-chip. The six influenza vaccine trial participates’ were stratified into high and low responders based on their clinical serum IgG fold changes (Fig. 7B). Using the chip model, we first confirmed these tested high-response donors exhibited significantly higher influenza-specific IgG secretion (Extended Data Fig. 5A), stronger DC/T/B activations (Extended Data Fig. 5B), and superior lymph node immune score (Extended Data Fig. 5C) than low responders in response to vaccine. We demonstrated that both high-dose vaccination (4× antigen) and IL-2 cytokine adjuvant treatments showed substantial improvements in most trial participates’ vaccine immunogenicity capacity and antibody outputs (Fig. 7C). These immunomodulatory interventions overall increased DC maturation (CD86), T cell (CD25) and B cell activation (CD69) compared to the standard vaccination condition (Fig. 7D, Extended Data Fig. 5D–G). These increases were accompanied by elevated T_fh_/T_fr_ ratios (Fig. 7E), indicating stronger helper T cell activity and reduced regulatory suppression. Cytokine profiling further supported robust B cell related humoral activation of IL-4, IFN-γ, and IL-8 under both conditions (Fig. 7F). B cell subset profiling confirmed progressive differentiation from naïve and pre-GC states toward GC and plasma cell phenotypes following high-dose vaccination and IL-2 cytokine adjuvant treatment (Extended Data Fig. 5H,I).

Correlation analyses validated synchronized immune reinforcement in the DC-T-B cascade: enhanced DC activations were associated with higher T cell activations (Fig. 7G), which in turn correlated to elevated B cell activations (Fig. 7H); elevated influenza-specific antibody secretions closely tracked with the increases in plasma cell frequency in the lymph nodes (Fig. 7I). Trail participates showed varied responses to these immunomodulatory interventions (Extended Data Fig. 5), whereas these intervention strategies overall showed more effect on low responders than high responders (Fig. 7C). While our results demonstrated that IL-2–driven STAT5 activation effectively expanded T cells, it did not consistently improve T_fh_ outputs across donors (Fig. 7E). This suggests that IL-2 alone might not be sufficient to optimize T_fh_–B cell cooperation in the lymph node, consistent with prior reports that excessive or unbalanced IL-2 signaling can restrain T_fh_ integrity and GC activity (45). Together, these mechanisms provide a mechanistic framework for personalized, pathway-targeted strategies to enhance vaccine responses in immunocompromised individuals.

## DISCUSSION

In clinical practice, vaccine efficacy is primarily evaluated by measuring serum antibody titers, an approach that lacks mechanistic insight into immunogenic potency (3, 4). Animal models, while the preclinical gold standard for preclinical evaluation of vaccines, are limited in their ability to predict human immune responses due to interspecies variation. Although humanized mouse models have been developed, physiological differences such as reduced and immature immune cell populations in lymph nodes, hinder accurate simulation of human adaptive immunity in human lymph nodes (46). To address this, we developed a personalized, immunocompetent human lymph node model that faithfully recapitulates the complex tissue architecture and dynamic immune functions of a native lymph node. The system successfully recapitulates the entire coordinated sequence of a vaccine-induced adaptive immune response: antigen capture and DC maturation in the paracortex, antigen-specific T cell activation and migration, T_fh_ cell generation, T-B cell collaboration in follicles, GC formation, and the production of antigen-specific antibodies. Thus, this model serves as an accurate in vitro proxy for human vaccine immunogenicity, enabling the evaluation of novel vaccine candidates with early, human-relevant efficacy and mechanistic data.

Human immune heterogeneity presents a significant challenge for developing effective vaccines across diverse populations. In parallel with an influenza vaccine trial, we reconstructed autologous lymph node models that recapitulates donor-specific immune heterogeneity using primary immune cells from individual trial participants. The vaccine clinical-trial-on-chip study replicates the known clinical impact of age and chronic health conditions, showing blunted vaccine responses in elderly and comorbid individuals. The magnitude of antibody production and cellular activation metrics from the chip strongly correlate with serum antibody titers measured from the same donors in vivo. More importantly, our study links the donor-specific immune heterogeneity to specific cellular and molecular signatures, e.g., a hyperactivated/exhausted baseline state and shift in DC–T_fh_–B cell axis in high-risk donors, offering insights into the biological basis of immune aging and comorbidity-associated dysfunction. By integrating orthogonal immune features (e.g., antibody secretion, DC/T/B cell activation, T_fh_/T_fr_ balance, GC and plasma cell composition) into a composite immune score, we established a quantitative, multi-parametric biomarker that correlates with clinical serology and directly links clinical risk to mechanistic immune readouts. This establishes our platform not merely as an in vitro model, but as a predictive proxy for a personalized evaluation of vaccine efficacy.

To translate cellular insights into actionable design principles, we applied scRNA-seq to pinpoint the key mechanistic bottlenecks underlying poor vaccine responsiveness in clinically vulnerable populations. Single-cell transcriptomic profiling of donor-specific immune signaling networks revealed three critical signaling nodes involving MyD88 in DCs, IL-2/STAT5 balance in T cells, and TACI/BCMA activation in B cells that collectively govern the efficiency of the adaptive immune cascade. Upon vaccination, DCs act as the primary sensors and initiators of adaptive immunity through Toll-like receptor (TLR)–MyD88 signaling, promoting antigen uptake, processing, presentation, and cytokine release (e.g. IL-6 and type I interferons) that guide lymphocyte activation (37). These signals instruct naïve CD4^+^ T cells toward T_fh_ differentiation through a finely-tuned balance between IL-2/STAT5 and IL-6/STAT3 pathways, as excessive IL-2 signaling suppresses BCL6 and limits T_fh_ commitment (39, 40). On the humoral side, the APRIL–TACI/BCMA axis supports plasma cell survival and long-term antibody secretion, completing the DC–T–B communication loop that defines effective vaccine responses (41). In low responders, bottlenecks emerged at each checkpoint: attenuated MyD88 signaling and weaker DC maturation; excessive IL-2/STAT5 activity without compensatory STAT3/ICOS signaling, limiting T_fh_ quality; and incomplete induction of plasma cell differentiation modules despite active PI3K–AKT proliferation signatures. These deficiencies explained their reduced GC activity and minimal antibody secretion. This provides an unprecedented, deep molecular understanding of the mechanisms driving variance in vaccine responsiveness, particularly within clinically vulnerable populations. Therefore, targeted interventions addressing these bottlenecks can serve as viable strategies to enhance vaccine efficacy.

Guided by these insights, we conducted a “clinical-trial-on-chip” study for screening clinically actionable vaccination strategies to enhance vaccine immunogenicity in immunocompromised individuals by selectively reinforcing these failure points. We demonstrated that high-dose vaccination amplified DC antigen presentation, IL-2 cytokine adjuvant enhanced T cell expansion. These targeted interventions partially restored coordinated DC–T–B signaling in low responders and markedly improved antibody production, illustrating how transcriptome-informed modulation can correct donor-specific immune deficits. In conclusion, the lymph node-on-a-chip platform presented here offers a powerful preclinical tool for studying human adaptive immunity, underscoring its potential of this approach in translational vaccine development and personalized immunization strategies.

## MATERIALS AND METHODS

### Human cohorts

Human PBMC and serum samples were obtained from the Stop Flu influenza vaccine clinical trial study New York University (NYU) cohort (clinical study identifier: S21-01215) during the 2022–2023 season. Participants donated blood before vaccination and approximately one month after vaccination with the seasonal influenza vaccine. Plasma was isolated by centrifugation, and PBMCs were isolated using Ficoll-Paque PLUS (GE Healthcare) or Sepmate (Stem Cell) tubes. Serum samples were stored at −80°C and PBMC samples were stored at liquid nitrogen until use. Human mesenteric lymph nodes were obtained from patients undergoing gastrointestinal surgery at NYU Langone Health. All procedures performed in studies involving human participants were approved by the Institutional Review Board (IRB) and conducted in accordance with the principles of the Declaration of Helsinki. All participants provided written informed consent. All of the samples were deidentified before analysis. The anonymous demographic information (age, gender, ethnicity) of these vaccine clinical trial participants is summarized in **Supplementary Table S1**.

### Cell culture

Human PBMC-isolated DCs, T cells, and B cells from healthy donors (iXCells Biotechnologies) and from influenza vaccine trial participants before vaccination at NYU Vaccine Center were used to build the lymph node immune niche. Specifically, human CD14^+^ monocytes, naïve B cells, B cells, and CD4^+^ T cells were isolated from PBMCs using magnetic-activated cell sorting (MACS) kits (StemCell Technologies). PBMC-isolated monocytes were then differentiated into imDCs via treating with 400 ng/mL GM-CSF (Miltenyi Biotec) and 250 ng/mL IL-4 (Miltenyi Biotec) in complete RPMI 1640 medium (Gibco) for 6 days. Prior to on-chip loading, fresh or frozen immune cells were thawed and recovered overnight in RPMI 1640 medium (Gibco) supplemented with 10% fetal bovine serum (FBS, Gibco), 1% penicillin/streptomycin (Gibco) in a 37 °C, 5% CO_2_ incubator. Human FRCs (Sciencell) were cultured in Fibroblast Medium (Sciencell) and used for generating a fibroblastic reticular network in the paracortex on chip. Primary human umbilical vein endothelial cells (HUVECs, Lonza) and RFP-expressing HUVECs (Angio-Proteomie) were cultured in were cultured in Endothelial Cell Growth Medium-2 BulletKit (EGM-2, Lonza), and used for generating vascular networks resembling HEVs within the paracortex. Human FDC line (KY6-CD40Lg-IL21 (47)) supporting follicular organization was obtained from Cell Bank at RIKEN BioResource Research Center (Tsukuba, Japan) and cultured in the RPMI 1640 medium (Gibco) supplemented with 10% FBS (Gibco). All primary stromal cells were used for experiments between passages 3 and 8.

### Vaccines

The Fluzone^®^ Quadrivalent Influenza Vaccine, 2022–2023 Formula (Cat#NR-59616) and 2017–2018 Formula (Cat#NR-51401) were obtained through Biodefense and Emerging Infections Research Resources Repository (BEI Resources, NIAID, NIH) for on-chip vaccination. Each 0.5 mL vaccine syringe contained 15 μg of the HA antigens of split-virus vaccine in PBS. For on-chip vaccination, vaccine was diluted 1:5000 in culture medium, yielding a final HA concentration of 6 ng/mL. The Fluzone^®^ Quadrivalent Influenza Vaccine (2022–2023 Formula) was used for chips derived from clinical trial participants. This vaccine has HA components that overlap with those used in the original clinical trials (see **Supplementary Table S2**).

### Microfluidic lymph node chip fabrication

The lymph node chip was designed with the paracortical chamber (for DCs, T cells, FRCs, and HUVECs) and the B cell follicle chambers (for B cells and FDCs), and the surrounding microfluidic channels mimicking subcapsular sinus, afferent vessels and efferent vessels that allow perfusion of fluids and antigens into the lymph node niche. The B cell follicle regions were partitioned from the paracortical region by regularly spaced PDMS micropillars (diameter 150 μm, interdistance 100 μm). These culture chambers were linked to three medium reservoirs to facilitate medium exchange and waste removal. The microfluidic chip was fabricated using a standard soft lithography technique by replica molding of PDMS (Sylgard184, Dow Corning) from a photolithographically prepared master mold on a glass coverslip (22 × 22 mm, Corning). The fabricated master mold was treated with perfluorooctyl trichlorosilane (Sigma-Aldrich) to ease release of PDMS replica from the mold. Once PDMS replica was made from the master mold, 1 mm, 1.5 mm, and 4 mm holes were punched for two side inlets, one central inlet, and four outlets, respectively, before it is bounded to the coverslip. Prior to cell loading, microfluidic chips were treated under ultraviolet for sterilization in a Type 2 class laminar flow hood for 20 minutes.

Cells were embedded in a biomimetic hydrogel composed of fibrin (6 mg/mL, Sigma-Aldrich), Matrigel (Corning), and type I collagen (3 mg/mL, Corning) at a ratio of 6:2:2 ratio before cell loading. T cells (5×10^7^ cells/mL), FRCs (5×10^6^ cells/mL), DCs (2×10^6^ cells/mL), and HUVECs (2×10^7^ cells/mL) were seeded into the paracortical chamber; B cells (5×10^7^ cells/mL) and FDCs (2×10^6^ cells/mL) into the B cell follicle chambers. After cell loading, the chips were cultured in a with a mixture of EGM-2 (Lonza), Fibroblast Medium (Sciencell), and RPMI 1640 (Gibco) at a 2:1:1 ratio and supplement with B cell activating factor (BAFF, 20 ng/mL Biolegend), Interleukin-4 (IL-4, 10 ng/mL, Miltenyi Biotec), and recombinant human VEGF-165/VEGF-A (10 ng/mL, RayBiotech), refreshed daily for optimal cell viability and function. Vaccines were added to the culture medium starting on day 1 post-seeding and refreshed daily to induce the vaccine immunogenicity response.

### Immunofluorescence staining and microscopy

Chips were fixed with 4% paraformaldehyde (Alfa Aesar) in PBS for 1 hour at 4 °C and then washed three times with PBS, 10 minutes each. For intracellular staining requiring membrane permeabilization (e.g., BCL6 and Ki-67), chips were incubated with 0.05% Triton X-100 (Sigma-Aldrich) in PBS for 20 minutes at room temperature, followed by PBS washes. Primary antibodies were diluted in staining buffer (Staining Buffer Set, BioLegend), supplemented with human TruStain FcX^™^ (BioLegend; 1:100 dilution) to block Fc receptors in immune cells, and 4′,6-diamidino-2-phenylindole (DAPI, 1 μg/mL, Thermo Fisher Scientific) to stain nuclei. Chips were incubated with the FITC-conjugated primary antibody/DAPI mixture overnight at 4 °C in the dark. For staining CCL19, chips were first incubated with unconjugated anti-CCL19 antibody, followed by a fluorophore-conjugated secondary antibody for 1 hour at room temperature in the dark, then washed with PBS before imaging. After staining, the chips were kept in Cell Staining Buffer (BioLegend) and imaged using a Nikon CSU-X1 Spinning Disk Confocal System, with subsequent image analysis performed using a Nikon NIS-Elements Microscope Imaging Software (Ar version). The fluorescence intensity of each maker was quantified using ImageJ (NIH) or Fiji (version 2, NIH).

The following markers were used to identify specific cell populations and functional states of cells on chip. Endothelial cells were identified by CD31, FRCs were identified by Podoplanin (PDPN), perivascular FRCs were identified by CD157 and secretory function assessed by CCL19 staining, and FDC line were identified by CD55. DCs were identified by CD11c, and their activation state was assessed by CD86 expression. T cells were identified by CD3, with CD4 and CD8 used in separate panels to define helper and cytotoxic subsets, respectively. T cell activation was assessed by CD25 or CD69 expression on CD3^+^ T cells. T_fh_ cells were identified as CD4^+^CXCR5^+^ and T_fr_ cells were identified by CD4^+^CXCR5^+^FOXP3^+^. B cells were identified by CD19, and activation was assessed by CD25 expression. GC structures were identified using CD19, BCL6, Ki67, and IgD: the whole GC area was identified by CD19^+^BCL6^+^ cells, the proliferative GC B cells in the dark zone were identified by CD19^+^Ki67^+^IgD^−^ cells, the non-proliferative light zone was identified by CD19^+^Ki67^−^IgD^+^ cells. The information about the antibodies and dilution conditions are listed in the **Supplementary Table S4**.

### Cytokine analysis

Culture supernatants were collected at a certain time point from chips, centrifuged at 2,000× g for 10 minutes at 4°C to remove cellular debris, and stored at −80°C for later cytokine measurements. Qualitative profiles of cytokine secretion were examined by using a Human Immune Response Array C1 membrane kit (RayBiotech) according to the manufacturer’s protocols. The membranes were imaged with a ChemiDoc Imaging System (Bio-Rad), and the mean intensity of each spot was quantified in ImageJ (NIH) using the Protein Array Analyzer plugin (developed by Gilles Carpentier, Faculté des Sciences et Technologies, Université Paris, Paris, France). Quantitative cytokine analyses were performed with respective enzyme-linked immunosorbent assay (ELISA) kits (BioLegend) to measure cytokine concentrations, following the manufacturer’s protocols using a UV-Vis spectrometer microplate reader (Sigma Aldrich).

### Influenza-specific antibody measurement

To assess influenza-specific antibody secretion levels in on-chip cultures and matched clinical serum samples, we used a custom-designed, bead-based multiplex influenza immunoassay on the Luminex^™^ platform (**Supplementary Methods**), which we ran in a 26-plex comprising 23 recombinant HA and NA antigens derived from historical and concurrent influenza A and B virus strains, complemented with Rubella Virus E1/E2 (ACROBiosystems) and BSA (Fisher Scientific) negative control antigens, as well as IC45 uncoupled, autofluorescing positive control beads (Luminex, MagPlex RP1 Monitor beads) (**Supplementary Table S3**). Fold change in IgG response was computed for each donor by dividing the post-vaccination signal by the corresponding pre-vaccination baseline.

Influenza-specific antibody levels in chip culture supernatants were also analyzed by ELISA following a standard protocol. ELISA plate (Corning) was pre-coated overnight at 4°C with 0.1 μg of seasonally corresponding Fluzone quadrivalent inactivated influenza vaccine (according to the total HA content as specified by the manufacturer) to serve as the capture antigen. Culture supernatants, diluted at a ratio of 1:20 with ELISA Assay Diluent (Biolegend), were then applied to these coated wells and incubated for 2 hours at room temperature. Horseradish peroxidase-labeled anti-human IgM/IgG/IgA (Abcam) were used as detection antibodies and incubated at room temperature for 1 hour to identify antigen-bound immunoglobulins. The plates were treated with 3,3′,5,5′-Tetramethylbenzidine (TMB) substrate solution (Thermo Scientific) for 15 minutes at room temperature in the dark, followed by the addition of 2 M sulfuric acid to stop the reaction. Lastly, optical density of each detection spot was measured at 450 nm by UV-Vis spectrometer microplate reader (Sigma-Aldrich), for a qualitative analysis of vaccine-specific antibody secretion in the chip.

### Statistics and reproducibility

Details regarding biological and technical replicates, as well as repetitions for each experiment, are listed in the respective figure legends. All error bars in the graphs are shown as mean and standard deviation of the mean (s.e.m.). Representative images shown in each figure are from one of three technical replicates with similar results. Significant differences between two groups were determined by unpaired two-tailed Student’s t-test with GraphPad Prism software (version 10), multiple groups were compared by one-way or two-way analysis of variance (ANOVA). Statistical analyses for flow cytometry data were performed in GraphPad Prism software (version 10.1.1). Statistical analyses for scRNA-seq were performed in R and are described in the relevant methods section and figure legends.

## Extended Data

## Supplementary Material

Supplementary Files

This is a list of supplementary files associated with this preprint. Click to download.

- SI20260223.pdf

## Data Availability

All data associated with this study are present in the paper or the Supplementary Materials. RNA-seq raw data reported in this paper are deposited in the Gene Expression Omnibus with accession number GSE293390. The Ensemble hg38/GRCh38 reference genome for scRNA-seq read alignment is available on NCBI Datasets (GCF_000001405.40).
